# A Cell-based Fluorescence Resonance Energy Transfer (FRET) Sensor Reveals Inter- and Intragenogroup Variations in Norovirus Protease Activity and Polyprotein Cleavage*

**DOI:** 10.1074/jbc.M115.688234

**Published:** 2015-09-11

**Authors:** Edward Emmott, Trevor R. Sweeney, Ian Goodfellow

**Affiliations:** From the Division of Virology, Department of Pathology, University of Cambridge, Addenbrookes Hospital, Hills Road, Cambridge CB2 2QQ, United Kingdom

**Keywords:** FRET, protease, RNA virus, substrate specificity, virus, calicivirus, norovirus, polyprotein

## Abstract

The viral protease represents a key drug target for the development of antiviral therapeutics. Because many protease inhibitors mimic protease substrates, differences in substrate recognition between proteases may affect their sensitivity to a given inhibitor. Here we use a cell-based FRET sensor to investigate the activity of different norovirus proteases upon cleavage of various norovirus cleavage sites inserted into a linker region separating cyan fluorescent protein and yellow fluorescent protein. Using this system, we demonstrate that differences in substrate processing exist between proteases from human noroviruses (genogroups I (GI) and II) and the commonly used murine norovirus (MNV, genogroup V) model. These altered the cleavage efficiency of specific cleavage sites both within and between genogroups. The differences observed between these proteases may affect sensitivity to protease inhibitors and the suitability of MNV as a model system for testing such molecules against the human norovirus protease. Finally, we demonstrate that replacement of MNV polyprotein cleavage sites with the GI or GII equivalents, with the exception of the NS6–7 junction, leads to the production of infectious virus when the MNV NS6 protease, but not the GI or GII proteases, are present.

## Teaser

**Background:** Noroviruses generate the viral replicase through polyprotein cleavage by a viral protease.

**Results:** A cell-based FRET sensor reveals significant variations in substrate cleavage both within and between norovirus genogroups.

**Conclusion:** Norovirus proteases from different genogroups vary in their substrate recognition.

**Significance:** Understanding the cleavage preferences of viral proteases is critical for drug design and provides insights into the potential for generating human-murine norovirus chimeras.

## Introduction

Noroviruses, members of the *Caliciviridae* family, represent the major cause of acute viral gastroenteritis in man and have also been observed in other animals, including dogs, cattle, and sheep. The *Norovirus* genus is divided into different genogroups, with human noroviruses (HuNoV)^3^ being found in G I, II, and IV, whereas murine norovirus (MNV) is the sole member of GV (1). MNV has, to date, provided the only robust norovirus model system that combines efficient cell culture replication with versatile reverse genetics systems and a homologous challenge animal model (2, 3). As a result, MNV has been used widely to study the intracellular life cycle of noroviruses (reviewed in Ref. 4). However, recent work has led to the observation that HuNoV can replicate, albeit to relatively moderate levels, in immortalized human B cells (5) and in immunocompromised mice (6). These latter experimental tools pave the way toward experimental systems to enable a detailed understanding of the HuNoV life cycle.

**FIGURE 1.**
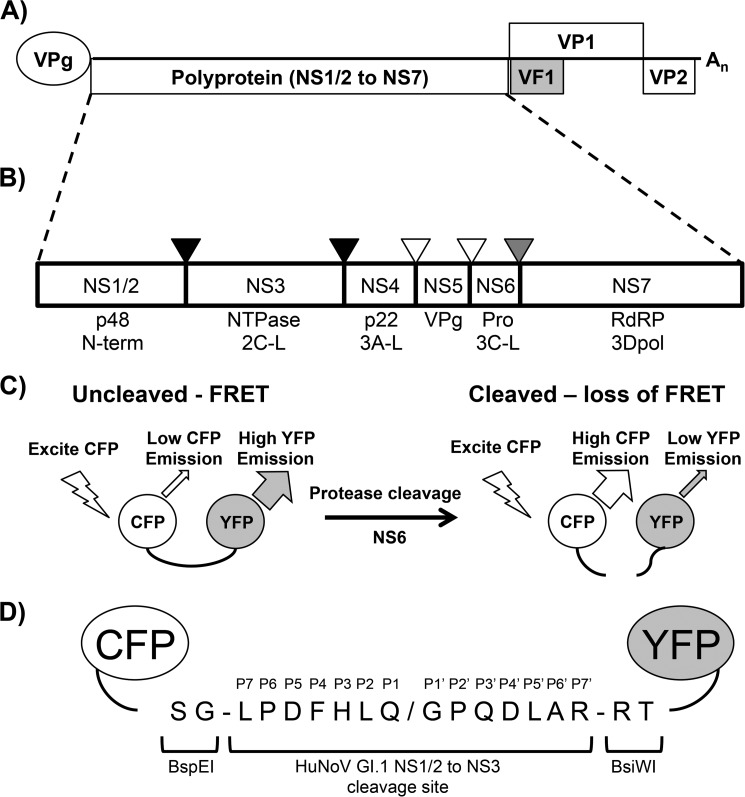
**Processing of the norovirus polyprotein and the principles underlying FRET sensors.** *A*, the norovirus genome is an ∼7.5-kb positive-sense, single stranded RNA molecule covalently bound to a viral protein, VPg, at the 5′ end. The genome encodes a large polyprotein as well as two additional open reading frames encoding the major (*VP1*) and minor (*VP2*) capsid proteins. GV norovirus possesses an additional overlapping reading frame encoding VF1. *B*, the polyprotein is processed by the viral protease (NS6) into a total of six proteins plus some stable intermediates. Cleavage at the NS1/2-NS3 and NS3-NS4 junctions is efficient (*black triangles*), processing at the NS6-NS7 junction occurs at a reduced level (*gray triangle*), and cleavage at the NS4-NS5 and NS5-NS6 junctions proceeds at low levels (*white triangles*). *C*, while the FRET sensor remains uncleaved, a high YFP/CFP ratio is observed. Upon cleavage by the protease of interest, the YFP/CFP ratio observed for the FRET sensor decreases. *D*, the FRET sensor used in this study consists of CFP fused to the N terminus of YFP, separated by an 18-amino acid linker. The N-terminal two residues of the linker represent a BspEI restriction site and the C-terminal two residues a BsiWI restriction site. The remaining 14 amino acids represent the P7 to P7′ residues of the cleavage site under investigation.

Viral genomes, particularly those of small RNA viruses, typically have a limited coding capacity because of the error-prone nature of the viral replication machinery and the dimensions of the viral capsid. Therefore, almost all positive-sense, single-stranded RNA viruses, including noroviruses, employ a number of strategies to maximize their protein-coding capacity (7). One mechanism used commonly is to encode many of the viral proteins responsible for replication of the viral genome in the form of a single large polyprotein that, in the case of the noroviruses, is cleaved into at least six proteins plus a number of stable intermediates (Fig. 1, *A* and *B*) (4). This cleavage is accomplished by the viral protease NS6.

Studies to date on the cleavage of the norovirus polyprotein by the viral protease have been performed using a number of approaches, including *in vitro* assays using FRET peptides or Western blotting of infected cell lysates (8–11), with one recent description of a luciferase-based in-cell assay (12). Despite the availability of crystal structures for the GI HuNoV protease (13–15) and, more recently, MNV (16, 17), the fine details of substrate recognition remain poorly characterized. The NS6 protease is a 3C-like cysteine protease with residues His-30, Glu-54 (Asp-54 in MNV), and Cys-139 comprising the active site (16). One recent advance was the finding that the P4 to P2′ region (following the nomenclature of Schechter and Berger (18)) surrounding each cleavage site represents the key determinant behind substrate recognition (19). Despite this, the contribution of specific residues, particularly on the P′ side of the cleavage site, remains poorly understood (17). Variation in the ability of the different norovirus proteases to cleave a given substrate clearly has the potential to influence the susceptibility of said proteases to substrate-based small molecule inhibitors or cleavage of host factors (20). For this reason, we set out to characterize the cleavage of the norovirus polyprotein by the viral protease using a cell-based FRET sensor approach (Fig. 1, *C* and *D*) (21, 22). This yielded the observation that several polyprotein cleavage sites within the human or murine norovirus polyproteins are cleaved at markedly different levels by the HuNoV or MNV protease. Individual residues within the substrate or protease were identified that contributed to this variation in activity. We demonstrate that the FRET sensor results are borne out when different cleavage sites are introduced into the MNV genome by reverse genetics and also give insights into differences in cleavage at the NS6-NS7 junction between the noroviruses and other members of the *Caliciviridae.* The differences in substrate recognition between the norovirus proteases suggest that the MNV model should be used with caution when investigating protease inhibitors to target human norovirus replication. Overall, these results provide new insights into the substrate specificity of norovirus proteases and provide a useful experimental system with which to further characterize norovirus protease function and specificity.

## Experimental Procedures

### 

#### 

##### Cell Lines and Plasmid Constructs

The murine microglial BV-2 cell line was provided by Jennifer Pocock (University College London, London, UK). BSR-T7 cells (baby hamster kidney cells engineered to express T7 polymerase) were provided by Karl-Klaus Conzelmann (Ludwig Maximilian University, Munich, Germany). FRET assays were performed in HEK293T cells. Cells were maintained in DMEM containing 10% fetal bovine serum, penicillin (100 units/ml) streptomycin (100 μg/ml), and, in the case of BSR-T7 cells, 0.5 mg/ml G418. Penicillin and streptomycin were omitted for transfection experiments.

Plasmids expressing norovirus protease from GI.1 (M87661), GII.4 (DQ658413), or GV (DQ285629) were generated by cloning of the relevant protease into the XhoI and BamHI sites of pmCherry-C1 (Clontech). Active site mutants of all proteases were generated though a H30A mutation by QuikChange mutagenesis (Stratagene). A FRET construct was generated by PCR of a fragment containing the GI NS1/2–3 protease cleavage site N-terminal of YFP into pECFP-C1 (Clontech) using the forward (GAC GAG CTG TAC AAG TCC GGA CTG CCA GAT TTC CAT CTA CAG GGC CCC GAG GAC CTT GCC AGG CGT ACG ATG GTG AGC AAG GGC) and reverse (GAG CTC GAG ATC TGA GTC CGG ACT TGT ACA GCT C) primers on a pEYFP-C1 template. The PCR fragment was inserted into BspEI-digested pECFP-C1 using the Gibson assembly kit (New England Biolabs), generating pFRET GI NS1/2–3 and removing the original BspEI site in the process. This generated an expression cassette containing CFP at the N terminus connected to YFP by an 18-amino acid cleavable linker (Fig. 1*D*). The cleavable linker within this construct contained BspEI and BsiWI restriction sites at its N and C terminus, respectively (residues 1, 2, 17, and 18), allowing all subsequent FRET constructs to be generated by ligation of annealed and phosphorylated oligos into BspEI/BsiWI-digested pFRET GI NS1/2–3. Details of the primers used are available upon request.

##### FRET Assay

HEK293T cells were seeded into black Greiner Cellstar 96-well dishes (Sigma) at a density of 5 × 10^4^ cells/well in 100 μl of DMEM containing 10% fetal bovine serum. 3 h after seeding, cells were transfected with 0.1 μg of protease and 0.1 μg of the appropriate FRET construct (or an appropriate empty vector control) by Lipofectamine 2000 transfection according to the protocol of the manufacturer (LifeTech). The transfected cells were incubated at 37 °C in a humidified incubator providing 5% CO_2_ for 24 h. At 24 h, the medium was removed and replaced with PBS. Plates were then analyzed on a Spectramax i3 instrument (Molecular Devices) in fluorescence mode, with excitation set at 434 nm and reading emission at 477 nm (CFP) and 527 nm (YFP). Background fluorescence was determined from wells transfected with pCDNA3.1 and subtracted. The YFP/CFP ratio was used to determine FRET efficiency as described previously (21). For ease of comparison, cleavage was sometimes expressed as percent cleavage relative to cleavage of a GI NS1/2-NS3 substrate by GI protease control. Statistical analysis was performed by one-way ANOVA in GraphPad Prism 6.

##### Reverse Genetics and Virus Yield Determination

cDNA-based reverse genetics rescues of wild-type or mutant MNV were performed as described previously (23). In brief, BSR-T7 cells were infected with recombinant fowlpox-expressing T7 polymerase. 3 h post-infection, cells were transfected with plasmids containing the MNV genome under the control of a T7 promoter, either wild-type MNV (pT7 MNV 3′RZ), MNV containing a lethal mutation in the polymerase active site YGDD-YGSN (YGSN), or the various mutant MNV constructs. 48 h post-transfection, cells were either frozen at −80 °C for subsequent titer determination or processed for Western blot analysis as described below. Determination of virus yield was performed by 50% tissue culture infectious dose (TCID_50_) in BV-2 cells as described previously (24). Mutant MNV genomes for reverse genetics were generated by two-step PCR mutagenesis of the pT7 MNV 3′RZ template as described previously (23). A full list of primer details is available upon request.

##### Western Blotting

Antisera against viral proteins (NS7, VPg/NS5) were raised in-house. Anti-GAPDH was purchased from Ambion, and anti-GFP N-terminal from Sigma-Aldrich. IRdye680 and 800 secondary antibodies were purchased from LI-COR. Samples were lysed in radioimmune precipitation assay buffer (50 mm Tris-HCl (pH 8.0), 150 mm NaCl, 1 mm EDTA, 1% Triton X-100, and 0.1% SDS). Sample concentrations were determined by BCA assay (Pierce). Lysates were resolved by SDS-PAGE and transferred to nitrocellulose membranes. Blocking and staining steps were carried out in 5% nonfat dried milk in PBS containing 0.2% Tween 20 and inclusion of the appropriate primary or secondary antibody. Membranes were imaged through detection of far-red fluorescence on a LI-COR Odyssey imager.

##### Structure Comparison

A model of the human norovirus GII.4 strain (GenBank accession no. AB541320.1) NS6 protease was generated by submitting the protease amino acid sequence to the online SwissModel server (25) using the structure of the Norwalk virus protease (PDB code 4IMQ, chain A) as a template. The Norwalk virus crystal structure (PDB code 4IMQ, chain A) and the norovirus GII.4 model were aligned to the MNV protease crystal structure (PDB code 4ASH, chain A). Residues 179–183 of the MNV protease B chain, present in the asymmetric unit of the MNV protease crystal structure, were then superimposed on the Norwalk virus protease crystal structure and norovirus GII.4 model to show the position of the peptide binding clefts. All structure alignments and comparisons were performed using the PyMOL molecular graphics software (Schrödinger, LLC) using the standard program-defined parameters.

## Results

### 

#### 

##### FRET Sensor for Assay of Norovirus Protease Activity

**FIGURE 2.**
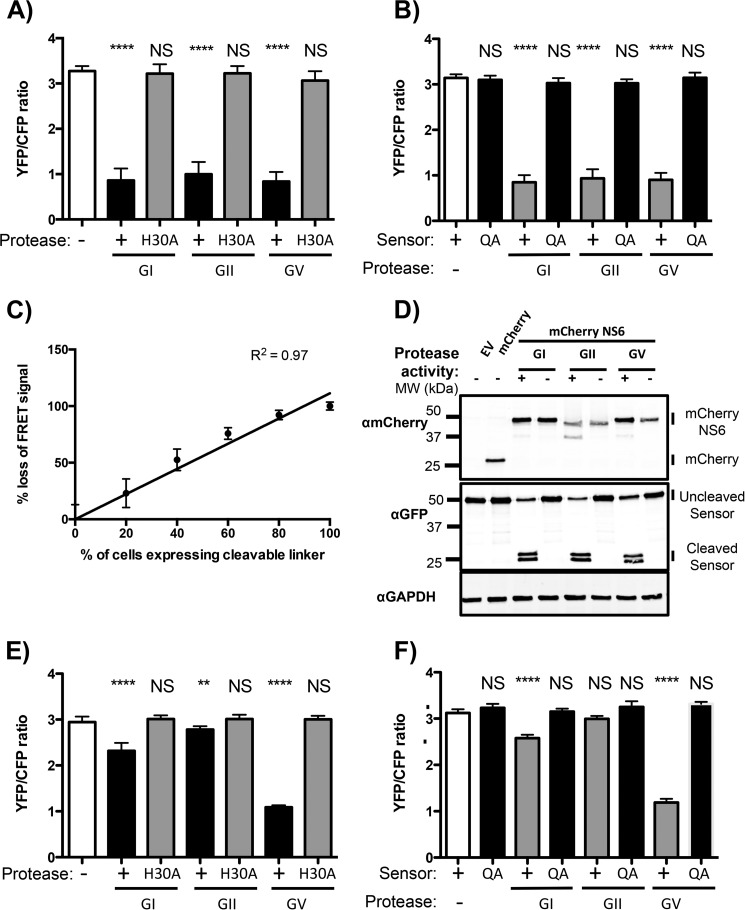
**Validation of FRET sensor function.** *A*, the FRET signal from a sensor on the basis of the GI NS1/2-NS3 cleavage site is lost upon incubation with active protease but not with inactive (*H30A*) protease. *NS*, not significant. *B*, the FRET signal is not lost when active protease is incubated with a FRET sensor where the P1 residue is mutated to alanine, rendering it non-cleavable. *C*, cells were transfected with protease and either cleavable or non-cleavable FRET sensor. 24 h post-transfection, the populations were mixed in defined ratios, and the FRET signal for each population was determined, revealing a linear relationship between the amount of cleaved FRET sensor in the sample and loss of FRET signal. *D*, Western blot analysis of cells cotransfected with GI NS1/2-NS3 FRET sensor and protease shows the appearance of cleavage products upon addition of active protease. *MW*, molecular weight; *EV*, empty vector. *E* and *F*, a FRET sensor on the basis of the GV NS1/2-NS3 cleavage site shows poor cleavage by GI or GII protease and is not cleaved by inactive protease (*E*) or when the P1 residue of the sensor is mutated to alanine (*F*). *Error bars* represent mean ± S.E. from a minimum of four biological replicates. Significance was determined by one-way ANOVA. *NS,* not significant. **, *p* < 0.01, ***, *p* < 0.001, ****, *p* < 0.0001.

To develop an in-cell assay for the detection of norovirus protease activity, a CFP-YFP fusion containing an 18-amino acid linker comprising an N-terminal BspEI site, C-terminal BsiWI site, and the P7 to P7′ residues from the GI.1 NS1/2-NS3 cleavage site was generated (pFRET GI NS1/2–3, Fig. 1*D*). This junction was chosen because previous data indicated that it can be cleaved efficiently by both the HuNoV GI and the MNV proteases (11, 26). The use of a FRET assay allowed the detection of cleavage in live cells. In the specific approach employed here, CFP was fused to the N terminus of YFP through a short linker region containing the P7 to P7′ residues of a potential substrate for the protease of interest. In the absence of cleavage, the CFP and YFP residues lie sufficiently close to allow FRET to occur, resulting in a high YFP signal but low CFP signal (Fig. 1*C*). Upon addition of a suitable protease, the linker is cleaved, separating the CFP from YFP molecules, resulting in the loss of YFP signal and a corresponding increase in CFP emission. This was measured on a plate reader by determining the YFP/CFP ratio. The FRET signal was observed in cells transfected with the sensor alone or when cotransfected with inactive protease containing a mutation in the catalytic site (H30A) (Fig. 2*A*). The YFP/CFP ratio matched that expected for a linker of this length (∼3.2) (21). An inability to cleave the sensor was also observed when the sensor was mutated to an alanine at the P1 residue (Fig. 2*B*). Because of limited information in the literature, the generation of substrates with defined levels of cleavage to determine the linearity of the assay was not possible. However, because the assay measures FRET within the population of transfected cells rather than on a single-cell basis, a substrate cleaved at a known efficiency (*e.g.* 50%) by the protease would give identical results to populations of cells transfected with either cleavable or non-cleavable sensor and mixed in defined ratios (*e.g.* 50:50). To test this hypothesis, cells were transfected with either the FRET sensor or the P1 alanine mutant and combined in defined ratios prior to assessing the FRET signal. The FRET signal decreased linearly with inclusion of increasing amounts of the cleavable FRET substrate (R^2^, 0.97) (Fig. 2*C*). Western blots were performed on samples containing active or inactive protease to confirm whether loss of FRET signal represented cleavage of the sensor. Cleavage products corresponding to CFP and YFP were detectable by anti-GFP Western blot on samples also transfected with active but not inactive protease (Fig. 2*D*). All FRET assays performed to this point used a substrate derived from the GI HuNoV polyprotein. To demonstrate that the assay would function with alternative substrates and could be used to distinguish between substrates that are cleaved efficiently and those that are not, an authentic murine norovirus sequence representing the P7 to P7′ residues of the GV NS1/2-NS3 cleavage site was also inserted into the FRET sensor (pFRET GV NS1/2–3). Unlike the GI.1 substrate, the GV NS1/2–3 sensor was cleaved inefficiently by the GI or GII proteases (Fig. 2*E*) and, as expected, inactive protease or P1 alanine mutants of the cleavage site (Fig. 2*F*). The MNV protease showed efficient cleavage of this substrate, whereas the GI protease showed only modest cleavage, and the GII protease displayed no significant evidence of cleavage. These data indicated that the assay could be used to assess variations in cleavage efficiencies between substrates.

##### Norovirus Protease Substrate Preferences Differ between Genogroups

**FIGURE 3.**
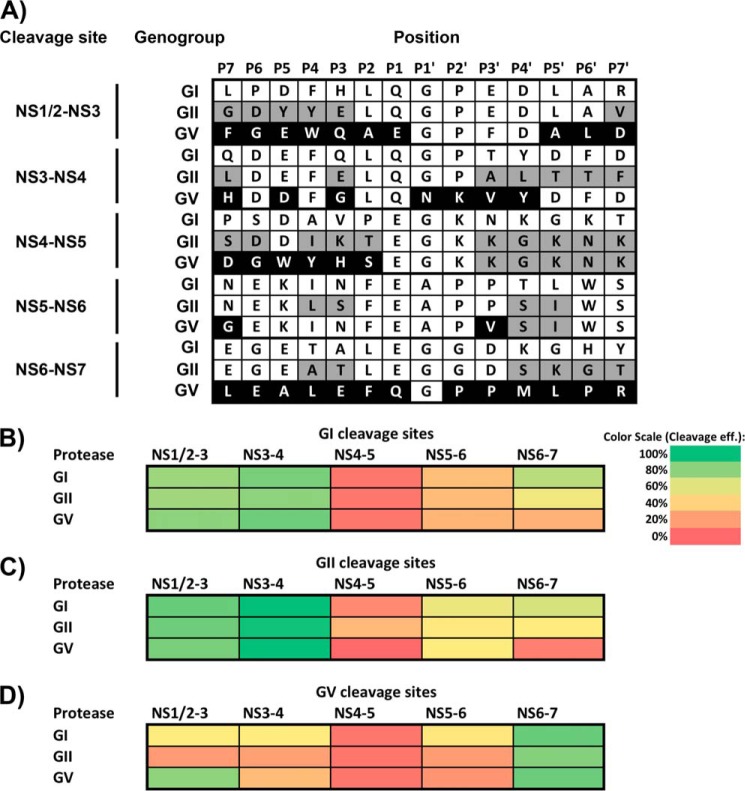
**FRET sensor analysis of GI, GII, and MNV polyprotein cleavage site processing by GI, GII, and MNV proteases.** *A*, polyprotein cleavage site sequences from GI, GII, and the MNV polyproteins. *Gray* and *black shading* highlights residues that differ from the GI, GII, and GV cleavage sites. *B*, cleavage of GI polyprotein cleavage sites by GI, GII, and MNV protease. *C*, cleavage of GII polyprotein cleavage sites by GI, GII, and MNV proteases. *D*, cleavage of GV polyprotein cleavage sites by GI, GII, and MNV proteases. Cleavage is shown on a *red* (low cleavage) to *green* (high cleavage) gradient and represents cleavage relative to cleavage of a GI NS1/2-NS3 control by GI protease. Each experiment was repeated a minimum of three times, and the average value was used. The *scale bar* illustrates colors at selected intervals of the gradient.

To gain a better understanding of divergence in the substrate recognition of the various norovirus proteases, the full complement of cleavage sites from the GI.1, GII.4, and MNV polyproteins were cloned into the FRET sensor to allow comparative analysis. Polyprotein cleavage of each genogroup has been tested previously *in vitro* and generally followed a pattern of efficient cleavage at the NS1/2-NS3 and NS3-NS4 junctions, moderate cleavage at the NS6-NS7 junction, and inefficient cleavage at the NS4-NS5 and NS5–6 junctions (Fig. 1*B*) (8–10). When each protease was assayed for the ability to cleave homologous substrates, *e.g.* GI protease on GI substrates, etc., as shown in Fig. 1*A*, a general pattern matching that obtained from the *in vitro* studies reported previously was observed (Fig. 3, *B–D*). However, cleavage at the NS3-NS4 site by the homologous proteases showed significant variation, with the MNV NS3-NS4 site showing markedly lower levels of cleavage than the equivalent sites from GI.1 or GII.4. Similarly, there was variation in the efficiency of cleavage observed at the NS6-NS7 sites by the homologous protease, with the MNV protease cleaving the homologous junction more efficiently than the GI and GII proteases.

When the various proteases were assayed on sensors representing the GI polyprotein cleavage sites, cleavage by GII or MNV protease largely matched that by the GI protease (Fig. 3*B*). However, cleavage of the NS6-NS7 site by MNV protease was impaired relative to the cleavage of this site by GI or GII protease. This same pattern was also observed when the various proteases were assessed against substrates representing GII polyprotein cleavage sites (Fig. 3*C*). Finally, upon cotransfection of the various proteases with FRET sensors representing GV polyprotein-based substrates, the poor cleavage of the NS1/2-NS3 cleavage site was again apparent (Fig. 3*D*). When assessing the cleavage of the GV polyprotein by MNV protease, a comparatively low cleavage of the GV NS3-NS4 site relative to its GI.1 and GII.4 equivalents was observed (Fig. 3*D*). The observation that this substrate is also cleaved at low levels by the GI or GII proteases suggested that this lessened cleavage was a feature of this particular substrate and not the GV protease. Neither the GI nor the GII protease exhibit any impaired cleavage of the GV NS6-NS7 substrate.

##### Sequence and Cleavage Efficiency of the GV NS3-NS4 Cleavage Site Varies within Genogroup V

**FIGURE 4.**
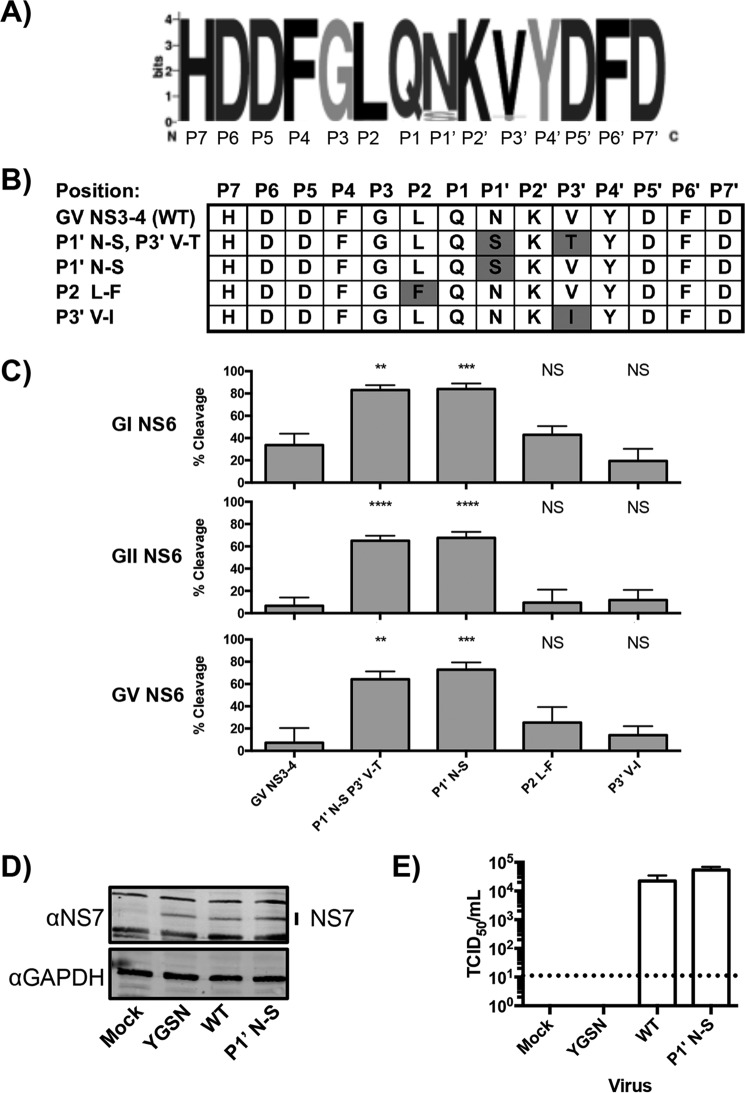
**Cleavage at the GV NS3-NS4 junction varies within the GV genogroup and is determined by the P1′ residue.** *A*, a sequence logo generated from 73 MNV sequences reveals that the GV NS3-NS4 FRET sensor conforms to the consensus sequence. *B*, the 73 sequences used to generate the sequence logo each correspond to one of five sequences. The variant sequences were used to generate novel GV NS3-NS4 FRET sensors containing the appropriate mutations. *C*, cleavage of FRET sensors containing a P1′ Asn-Ser mutation show enhanced cleavage, demonstrating a role for the P1′ residue. The *y* axis shows cleavage relative to cleavage of a control GI NS1/2-NS3 FRET sensor by GI protease. *Error bars* represent mean ± S.E. from a minimum of four biological replicates. Significance was determined by one-way ANOVA. *NS*, not significant. **, *p* < 0.01, ***, *p* < 0.001, ****, *p* < 0.0001. *D*, Western blot analysis of rescues of a P1′ Asn-Ser MNV mutant virus. *E*, titers obtained from rescues of a MNV P1′ Asn-Ser mutant virus. A *dotted line* highlights the assay limit of detection. *Error bars* represent mean ± S.D. from three biological replicates.

The cleavage sites from GV were derived from the prototype MNV strain CW1 (NCBI accession no. DQ285629) (23). To determine whether the lessened cleavage of this site was an unusual feature of this particular viral isolate, sequence logos were prepared by alignment of 73 full-length MNV polyprotein sequences (Fig. 4*A*). The sequence logo confirmed that the vast majority of these sequences conformed to the consensus sequence, including the sequence under assay. Only eight cleavage sites differed from the consensus (JQ237823, JF320652, JF320646, HQ317203, EU004677, EU004673, EU004676, and DQ911368), representing four divergent sequences in total (Fig. 4*B*). When the relevant sequences were introduced into the FRET sensor, substitution of the P1′ or P1′ and P3′ residues was sufficient to enhance cleavage to levels comparable with the NS3-NS4 sites from the GI.1 or GII.4 polyproteins (Fig. 4*C*). These data suggest that a serine represents a preferred P1′ residue in this context. Analysis of the cognate protease sequences from the MNV strains containing these divergent sequences failed to reveal any residues around the substrate binding site that might account for this variation (data not shown), suggesting that no compensatory mutation in the protease occurs. To confirm that the enhanced cleavage at the NS3-NS4 junction was tolerated during MNV infection and that no other compensatory mutations in NS6 were required for virus viability, reverse genetics was used to introduce a Asn-Ser mutation at the P1′ site of NS3–4. Western blotting of cells transfected with the infectious MNV cDNA plasmid, performed as a loading control, showed equivalent NS7 levels in each of the samples (Fig. 4*D*). Both the wild-type and the Asn-Ser mutant produced equivalent levels of infectious virus following reverse genetics rescue, whereas a polymerase active site mutant (YGSN) used as a negative control failed to yield any detectable virus (Fig. 4*E*). These data indicate that natural variation within a genogroup can lead to variation in cleavage efficiency at particular sites within the polyprotein. The divergent strains that have been phenotyped *in vivo* appear to all represent persistent strains (CR3, CR6, and CR7 (27)), raising the possibility that persistent or acute strains of norovirus may exhibit altered precursor levels.

##### Sequence Determinants of Cleavage at the GV NS1/2-NS3 Junction

**FIGURE 5.**
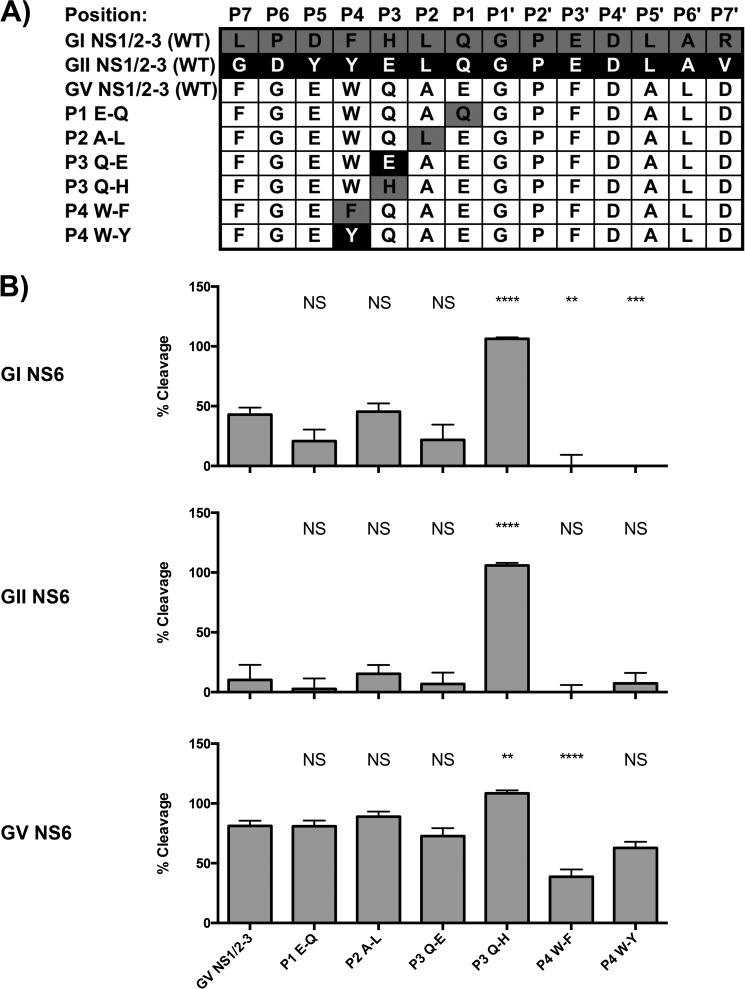
**Poor cleavage of GV NS1/2-NS3 by GI and GII proteases can be rescued by substitution of the P3 residue for histidine.** *A*, residues in the P4 to P1 region of the GV NS1/2-NS3 cleavage site were substituted for the equivalent residue from the GI or GII site. *B*, substitution of the P3 residue for histidine rescues cleavage of the GV NS1/2-NS3 site by GI or GII protease. The *y* axis shows cleavage relative to cleavage of a control GI NS1/2-NS3 FRET sensor by GI protease. *Error bars* represent mean ± S.E. from a minimum of four biological replicates. Significance was determined by one-way ANOVA. *NS*, not significant. **, *p* < 0.01, ***, *p* < 0.001, ****, *p* < 0.0001.

As observed in Figs. 2*E* and 3, the GI and GII proteases processed the GV NS1/2-NS3 cleavage site poorly. The cleavage sites at this position are highly divergent between genogroups (Fig. 5*A*). May *et al.* (19) have demonstrated recently that the P4 to P2′ sites comprise the core region of the cleavage site recognized by GI or GII human norovirus protease and are essential for determining the efficiency of cleavage. To further define the specific amino acids within this junction that contribute to cleavage efficiency, a series of mutants was constructed in which individual residues of the GV cleavage site were replaced by their equivalent residues from the GI.1 or GII.4 NS1/2-NS3 cleavage sites (Fig. 5*A*). Substitution of the P3 site for His, but not Glu, notably enhanced cleavage of this cleavage site by all three proteases. In the case of the GI and GII proteases, cleavage rose to levels equivalent to cleavage of their cognate NS1/2-NS3 substrates (Fig. 5*B*). Substitutions at the P1 and P2 sites had little effect, and substitutions at the P4 site showed some decrease in cleavage.

##### Comparison of Protease Structures Suggests a Role for Residue 110 in P3 Site Sensitivity

**FIGURE 6.**
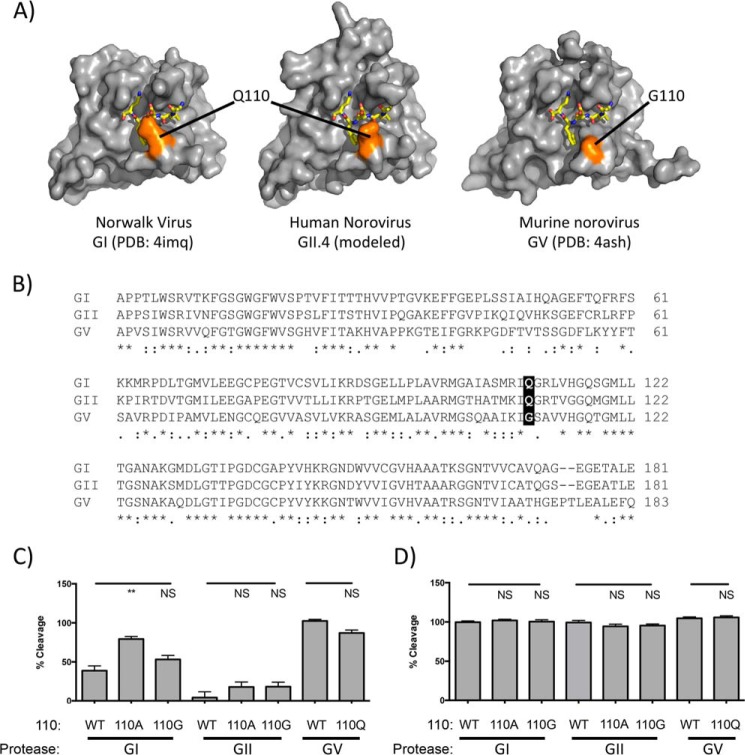
**Substitution of residue 110 enhances cleavage of the GV NS1/2-NS3 junction by GI and II proteases.** *A*, surface representations of norovirus protease structures. *Left panel*, Norwalk virus (PDB code 4IMQ; chain A, surface). *Right panel*, MNV (PDB code 4ASH; chain A, surface; chain B residues 179–183, sticks). *Center panel*, a model of the norovirus GII.4 protease generated in SwissModel (48) using the Norwalk virus protease as a template as described under “Experimental Procedures.” Residues 179–183 of a second molecule of the MNV protease present in the asymmetric unit (representing P5 to P1 of the NS6-NS7 cleavage site) are shown as *yellow sticks*. These residues were superimposed on the Norwalk virus structure and GII.4 protease model as described under “Experimental Procedures” to show the substrate-binding cleft. Residue 110 is highlighted in *orange. B*, sequence alignment of the GI, GII, and MNV proteases used in this study, with residue 110 *highlighted. C*, mutation of GI or GII residue 110 leads to enhanced cleavage of a GV NS1/2-NS3-based sensor. *D*, mutations at residue 110 do not inhibit cleavage of a control GI NS1/2-NS3 sensor. The *y* axis shows cleavage relative to cleavage of a control GI NS1/2-NS3 FRET sensor by the GI protease. *Error bars* represent mean ± S.E. from a minimum of four biological replicates. Significance was determined by one-way ANOVA. Structure images were generated in PyMOL (Schrödinger, LLC). *NS*, not significant. **, *p* < 0.01, ***, *p* < 0.001, ****, *p* < 0.0001.

Crystal structures of the NS6 proteases from Norwalk virus, Chiba virus and Southampton virus (GI) (13–15) and the MNV protease (GV) have been solved previously (16, 28). The structures of these proteases with partial substrates in the form of peptide inhibitors (14) or extended termini present in the substrate binding cleft (16, 28) have identified important determinants of substrate specificity. Importantly, although considerable information has been obtained about the recognition of key residues in the P1 region of the substrate, interactions between the protease and residues of the P1′ region have not been observed (17). Comparing the GI and GV protease structures using the PyMOL molecular graphics system (Schrödinger, LLC) (as described under “Experimental Procedures”), we identified an amino acid substitution between the two proteases that is positioned in close proximity to the P3 side chain of the peptide substrate (highlighted in *orange* in Fig. 6*A*, *left* and *right panels*). This residue, at amino acid position 110, is a Gln in the GI and GII proteases and a Gly in the GV protease (Fig. 6*B*). We generated a model structure of the human norovirus GII.4 NS6 protease using the SwissModel server (25), with the GI Norwalk virus protease as a template (Fig. 6*A*, *center panel*). In the model, the relative position of residue 110, which again is a Gln, is the same as for the GI and GV proteases. Residue 110 is part of a loop that forms one side of the S2 pocket. Considerable flexibility was observed in this loop and in Gln-110 in crystal structures of the Norwalk virus protease, with different peptide substrates in the binding cleft (28). Substitution of Gln-110 of the GI protease for Ala caused a significant increase in cleavage of the GV NS1/2 to NS3 substrate (Fig. 6*C*). A Q110G mutation of the GI protease or Q110G/A substitution of the GII protease also showed a trend toward increased cleavage activity on this substrate, but this was not statistically significant (Fig. 6*C*). All amino acid 110 mutants cleaved the GI NS1/2-NS3 FRET sensor substrate, a site cleaved robustly by all of the wild-type proteases, as efficiently as the wild-type proteases, confirming that the mutations did not induce a major defect in activity (Fig. 6*D*).

##### Insertion of Cleavage Sites of Comparable Cleavage Efficiency Is Tolerated in the Context of MNV Infection

**FIGURE 7.**
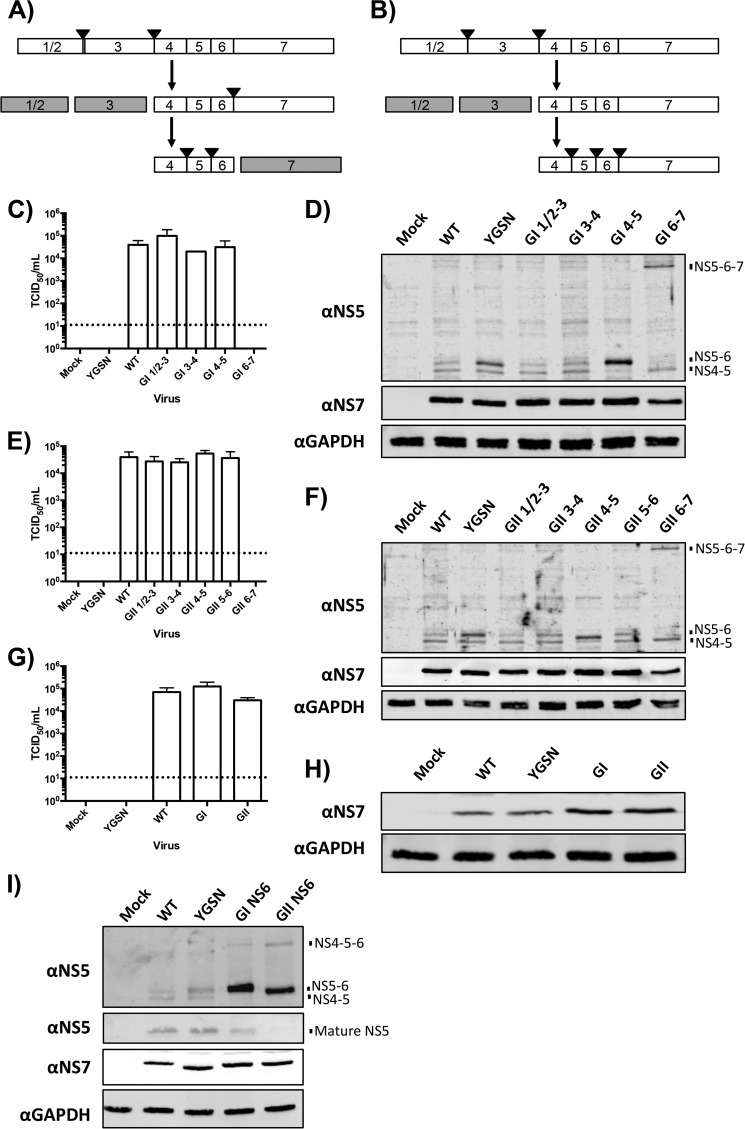
**Introduction of the GI or GII cleavage sites into the MNV genome is tolerated where the sites are processed at an equivalent level by the GV protease.** *A*, cleavage pattern of the MNV polyprotein containing an NS3-NS4 P1′ Asn-Ser mutation. *Black triangles* indicate NS6 cleavage. *B*, predicted GI polyprotein processing by the MNV protease. *C*, TCID_50_ of MNV containing substitutions of individual GI processing sites. *D*, Western blot analysis of MNV rescues containing substitution of individual GI processing sites. *E*, TCID_50_ of MNV containing substitutions of individual GII processing sites. *F*, Western blot analysis of MNV rescues containing substitution of individual GII processing sites. *G*, TCID_50_ of MNV rescues containing substitutions of all tolerated GI or GII protease cleavage sites. *H*, Western blot analysis of MNV rescues containing substitutions of all tolerated GI or GII protease cleavage sites. *I*, Western blot analysis of MNV rescues containing all tolerated cleavage site substitutions and replacing the MNV protease with the GI or GII protease. *Error bars* represent mean ± S.D. from a minimum of four biological replicates. The detection limit of the assay is indicated by a *dotted line*.

Data on cleavage of polyprotein substrates by the MNV protease have revealed that, although there may be variation in NS3-NS4 cleavage efficiency, modification of this site to yield the general pattern of polyprotein processing seen in the noroviruses with rapid processing at the NS1/2-NS3 and NS3-NS4 sites, slower processing at the NS6-NS7 site, and very slow processing at the NS4-NS5 and NS5-NS6 sites is well tolerated (Fig. 7*A*). However, when the cleavage sites within the MNV polyprotein were modified to those from the GI or GII polyprotein, the poor cleavage observed at the NS6-NS7 junction would be predicted to produce an aberrant processing pattern (Fig. 7*B*). The predicted cleavage of an MNV polyprotein including GI or GII cleavage sites would have vastly reduced processing at the NS6-NS7 cleavage site, leading to accumulation of NS6-NS7-containing precursors. The P4 to P2′ region of the cleavage site has been shown recently to be sufficient for substrate recognition by the norovirus protease (19), and, therefore, the P4 to P2′ residues of each of the GI polyprotein cleavage sites were introduced into the MNV polyprotein in the context of the full-length infectious cDNA clone (Fig. 7*C*). No substitution of the GI NS5-NS6 cleavage site was made because this site is identical in both the GI and MNV polyproteins. As predicted, substitutions of the GI NS1/2–3, NS3-NS4, and NS4-NS5 junctions were tolerated by MNV following reverse genetics rescue, whereas substitution of the NS6-NS7 site was not tolerated by MNV, reducing virus titers below the detection threshold, similar to the YGSN negative control (Fig. 7*C*). Western blot analysis of these cells transfected with the mutant cDNA constructs, where protein expression is driven by T7 RNA polymerase, revealed that cleavage at the NS6-NS7 junction was inefficient, although not ablated totally (Fig. 7*D*). Replacement of the NS6-NS7 junction caused the NS5-NS6 precursor to instead appear as an NS5-NS6-NS7 precursor (Fig. 7*D*). Substitution of the MNV polyprotein cleavage sites with their GII equivalent, including the NS5-NS6 cleavage site, was similarly tolerated in the context of MNV infection (Fig. 7*E*). As with substitution of the GI NS6-NS7 cleavage site, substitution with the GII NS6-NS7 cleavage site was lethal to MNV and resulted in the accumulation of an NS5-NS6-NS7 precursor (Fig. 7*F*). Of note, the insertion of either the GI or GII NS4-NS5 cleavage site into the MNV polyprotein appeared to induce a modest alteration in the pattern of NS5 precursors observed (Fig. 7, *D* and *F*). In both cases, increased accumulation of an NS5-NS6 precursor was observed alongside decreased accumulation of the NS4-NS5 precursor. The increased accumulation of the NS5-NS6 precursor possibly reflected enhanced cleavage of an NS4-NS5-NS6 precursor, a precursor that is not detectable by Western blot. The simultaneous introduction of all individual tolerated GI (NS1/2-NS3, NS3-NS4, and NS4–5) or GII (NS1/2-NS3, NS3-NS4, NS4-NS5, and NS5-NS6) cleavage sites into the MNV polyprotein was viable in the context of MNV infection (Fig. 7, *G* and *H*).

Finally, it was hypothesized that if cleavage sites within the MNV polyprotein were compatible with the GI or GII protease, then a HuNoV protease might be able to functionally replace the MNV protease. The GI and GII proteases were introduced into the combined cleavage site substitution viruses from Fig. 7*G* in place of the MNV protease. Although neither of these viruses were viable (data not shown), Western blot data confirmed that polyprotein processing occurred with efficient cleavage of the NS6-NS7 junction (Fig. 7*I*). Poor processing at the NS5-NS6 junction led to low levels of fully processed NS5 and increased accumulation of the NS5-NS6 precursor. Although levels of the NS4-NS5 precursor did not appear increased because of the low cleavage of the NS5-NS6 junction this precursor instead appeared as a higher molecular weight NS4-NS5-NS6 precursor.

## Discussion

Because the viral protease performs an enzymatic activity not seen in uninfected cells, it represents a key target for antiviral therapy. Here we demonstrate that a sensor based on loss of FRET signal upon cleavage by the norovirus protease represents a rapid and robust means to characterize protease function and examine protease specificity. In agreement with previous studies, cleavage of the various polyprotein-based FRET substrates followed the general pattern of efficient cleavage between the N-terminal NS1/2 and NS3 as well as the NS3 and NS4 proteins, with moderate cleavage observed between NS6 and NS7 and inefficient processing at the NS4-NS5 and NS5-NS6 cleavage sites (8–10). One exception to this pattern was the comparatively poor cleavage of the GV NS3-NS4 cleavage site by all proteases assayed. Subsequent analysis of variants of this cleavage site obtained from different MNV strains revealed that differences in the cleavage of this site between different stains were apparent, with some variations enhancing cleavage by GI, GII, and MNV proteases. The variant with the greatest effect on cleavage was at the P1′ position (Fig. 4), in agreement with the recent observation that the P4 to P2′ region is the core region necessary for substrate recognition (19). Previous structural investigations of the norovirus protease have revealed roles for residues on the P, but not the P′ side of the cleavage site (13, 16, 17), so knowledge of P1′ variants affecting, and tolerated for, cleavage may assist in elucidating the residues within the protease important for P′ recognition and specificity. Whether variation in polyprotein cleavage rates contributes to the variation in viral tropism and pathogenesis seen between isolates has yet to be determined and will require further study. However, there are precedents for this in other RNA viruses whereby controlling the relative levels of precursors can contribute to viral pathogenesis. One of the best studied examples is the accumulation of the NS2-NS3 precursor of bovine viral diarrhea virus, resulting in a non-cytopathic phenotype, inhibition of apoptosis, and the innate immune response (29).

The poor cleavage of the GV NS1/2-NS3 site by the GI or GII proteases was restored by modification of the P3 position. Previous structural analyses showed contacts between the P1, P2, and P4 residues and the protease but not the P3 position, consistent with the variable nature of residues found in this position in different cleavage sites (16, 28). May *et al.* (19) recently identified a role for the P3 residue in substrate recognition. In contrast to our study, they found that a His-Glu substitution in the context of a GI NS1/2-NS3 substrate enhanced activity, whereas our data showed no improvement on cleavage of a different GV NS1/2-NS3 substrate with a Gln-Glu substitution but a marked improvement with a Gln-His mutation. Our data suggest that the P3 position may be involved in more subtle alterations of the protease-substrate complex. Residues 109–112 of the protease constitute a loop that forms part of the S2 substrate-binding pocket and is in close proximity to the P3 residue. Although direct interactions with the P3 residue are not seen, it is possible that the substitution of the P3 residue could affect the organization or flexibility of this loop and thereby alter the S2 pocket. Indeed, Muhaxhiri *et al.* (28) have demonstrated that residues in this loop of the GI Norwalk virus protease were flexible, adopting different conformations depending on the sequence of the substrate. In contrast to the bulkier side chains that comprise the loop in the GI proteases, the loop of the GV MNV protease is more compact (Fig. 6). The more compact nature of the 109–112 loop in the GV protease may help to explain the enhanced activity against certain cleavage junctions. In support of this, our results show that a Q110G (or Ala) substitution within GI or GII proteases, as found in the GV protease, enhanced cleavage of the GV NS1/2-NS3 FRET sensor, otherwise cleaved poorly by the GI and GII proteases.

Variation in cleavage efficiency at the NS6-NS7 junction was observed consistently using the FRET-based sensor experimental system. In other members of the *Caliciviridae*, such as vesiviruses (30, 31) or sapoviruses (32, 33), the NS6(protease)-NS7(polymerase) precursor is not cleaved, despite the presence of a potential protease cleavage site. Clearly, in this form in norovirus, NS6 remains functional because other cleavage junctions are still processed normally, and these data are in line with previous observations on human norovirus Pro-Pol activity (34, 35). Efficient cleavage at this site in MNV represents a divergence from the general trend observed within the *Caliciviridae* family, and it has been shown previously that total ablation of cleavage at this site is lethal for viral replication (36). Expanding on these previous observations, we observed that even slight decreases in the cleavage efficiency of the NS6-NS7 site, as occurred when either the GI or GII equivalent cleavage sites were introduced, are lethal for MNV replication (Fig. 7, *C* and *E*). Modification of the NS6-NS7 cleavage site not only reduced the levels of mature NS7 produced but also lead to a complete loss of a detectable pool of the NS5-NS6 precursor, with this precursor instead appearing as a higher molecular weight NS5-NS6-NS7 precursor (Fig. 7, *D* and *F*). The role of precursors in RNA virus replication is becoming increasingly clear (reviewed in Ref. 37). For example, during poliovirus replication, the VPg-containing precursor 3BC, equivalent to the norovirus NS5-NS6 precursor, is nucleotidylated by the viral RNA-dependent RNA polymerase prior to linkage to the viral genome (38). Whether the lack of replication in viruses containing the GI or GII NS6-NS7 cleavage sites was due to the direct effect on the levels of the viral RNA polymerase, NS7, or the inability to produce a pool of the NS5-NS6 precursor is unknown and will require further study. These data may also indicate that, as also seen with poliovirus (38), processing of the NS6-NS7 junction may precede and be required for efficient cleavage of the NS5-NS6 junction.

Recombination between RNA virus genomes has been reported widely, and recombinant noroviruses have been observed circulating in the human population, including intergenogroup recombinants between GI and II (39–41). In the vast majority of cases, recombination occurs within genogroups and occurs at the ORF1/2 junction (42). However, recombination of viruses within the polyprotein region may also occur (43). From the data presented here, the GI and GII polyproteins appeared to be cleaved similarly, with both proteases showing similar activity on the heterologous substrates, suggesting that recombination within the polyprotein would not necessarily be restricted by differences in polyprotein cleavage. Recent work by Zhang *et al.* (44) on two MNV strains has demonstrated the generation *in vivo* of a variety of recombinants throughout the polyprotein region. Notably, the two strains used (CW3 and WU20) possessed highly similar cleavage sites. Attempts to generate HuNoV-MNV chimeras have, to date, been unsuccessful, with the data presented here suggesting that cleavage incompatibility may have been one factor that stymied these efforts. However, incompatibilities between the viral polymerase and capsid (45) or other viral proteins may also contribute.

The recombinant MNV viruses expressing GI or GII polyprotein cleavage sites were notable for the variation observed in precursor formation. When the NS4-NS5 junction was replaced with that from the GI or GII polyprotein, increased accumulation of the NS5-NS6 precursor was observed alongside diminished levels of the NS4-NS5 precursor (Fig. 7, *E* and *G*). This suggests that MNV replication can tolerate some variation in the levels of the various precursors. However, the diminished cleavage at the NS5-NS6 (Fig. 7*I*) or NS6-NS7 cleavage sites (Fig. 7, *D* and *F*) indicates that this tolerance has definite limits.

Finally, efforts to generate protease inhibitors against the norovirus protease have generally focused on small molecules that mimic peptide substrates of the protease (46, 47). Where these inhibitors have been assessed on both HuNoV replicons and MNV, some, often significant, divergence in their efficacy has been observed (48). Although differences in the norovirus replicon *versus* infectious systems used may partially explain some of these differences, our data suggest that differences in substrate recognition by the proteases themselves may also contribute.

Overall, this work provides insights into substrate recognition by the norovirus protease and into how differences in the protease can affect cleavage of polyprotein-based substrates. Our data suggest that substrate-based inhibitors of the norovirus protease should be assessed on human norovirus replicons, whereas data from MNV should be interpreted with caution, and that further work to assess the substrate specificity and differences in norovirus protease activity is warranted.

## Author Contributions

E. E. conceived the study. E. E. and T. S. designed, performed, and analyzed the experiments. E. E., T. S., and I. G. interpreted the data. E. E. and I. G. wrote the manuscript. All authors gave final approval of the manuscript for publication.

## Footnotes

3 The abbreviations used are:

HuNoV: human norovirus
MNV: murine norovirus
GI: genogroup I
ANOVA: analysis of variance
CFP: cyan fluorescent protein
YFP: yellow fluorescent protein
TCID_50_: 50% tissue culture infectious dose.
